# Common and Rare Variants in Genes Associated with von Willebrand Factor Level Variation: No Accumulation of Rare Variants in Swedish von Willebrand Disease Patients

**DOI:** 10.1055/s-0040-1718885

**Published:** 2020-10-31

**Authors:** Eric Manderstedt, Christina Lind-Halldén, Stefan Lethagen, Christer Halldén

**Affiliations:** 1 Department of Environmental Science and Bioscience, Kristianstad University, Kristianstad, Sweden; 2 Department for Coagulation Disorders, University Hospital, Malmö, Sweden; 3 National Hemophilia Center, Copenhagen University Hospital-Rigshospitalet, Copenhagen, Denmark; 4 Sobi, Stockholm, Sweden

**Keywords:** von Willebrand factor, von Willebrand disease, rare variant, DNA sequencing

## Abstract

Genome-wide association studies (GWASs) have identified genes that affect plasma von Willebrand factor (VWF) levels. *ABO* showed a strong effect, whereas smaller effects were seen for *VWF* , *STXBP5* , *STAB2* , *SCARA5* , *STX2* , *TC2N* , and *CLEC4M* . This study screened comprehensively for both common and rare variants in these eight genes by resequencing their coding sequences in 104 Swedish von Willebrand disease (VWD) patients. The common variants previously associated with the VWF level were all accumulated in the VWD patients compared to three control populations. The strongest effect was detected for blood group O coded for by the *ABO* gene (71 vs. 38% of genotypes). The other seven VWF level associated alleles were enriched in the VWD population compared to control populations, but the differences were small and not significant. The sequencing detected a total of 146 variants in the eight genes. Excluding 70 variants in *VWF* , 76 variants remained. Of the 76 variants, 54 had allele frequencies > 0.5% and have therefore been investigated for their association with the VWF level in previous GWAS. The remaining 22 variants with frequencies < 0.5% are less likely to have been evaluated previously. PolyPhen2 classified 3 out of the 22 variants as probably or possibly damaging (two in *STAB2* and one in *STX2* ); the others were either synonymous or benign. No accumulation of low frequency (0.05–0.5%) or rare variants (<0.05%) in the VWD population compared to the gnomAD (Genome Aggregation Database) population was detected. Thus, rare variants in these genes do not contribute to the low VWF levels observed in VWD patients.

## Introduction

The von Willebrand factor (VWF) is a multimeric glycoprotein that has a key role in hemostasis and shows widely varying plasma levels in the normal population.

Quantitative deficiencies in VWF arise from changes in its biosynthesis, secretion, and clearance. 1 Low VWF levels are associated with an abnormal bleeding phenotype, von Willebrand disease (VWD). Type 1 VWD is the least serious type, accounting for approximately 70% of diagnosed cases. It is defined as a partial deficiency of functionally normal VWF and in many cases shows dominant inheritance. Most severe cases of type 1 VWD are caused by mutations in the *VWF* gene, and often patients with moderately reduced levels of VWF do not have a mutation in the *VWF* gene. 2 A previously published genome-wide association study (GWAS) identified eight genes that contribute to plasma level variation of VWF. 3 The *ABO* gene showed the strongest effect, but smaller effects were seen for the *VWF* , *STXBP5* , *STAB2* , *SCARA5* , *STX2* , *TC2N* , and *CLEC4M* genes. Several exome-wide association studies and GWASs have since investigated the contribution of nucleotide variation to plasma level of VWF and replicated the aforementioned results. 4 5 6 The study by Huffman et al 4 used chip-based genotyping of common and low-frequency variants and identified additional loci within previously reported genes that had effect sizes much larger than, and independent of, previously identified common variants. A rare *STAB2* variant (rs141041254) had an effect size on the VWF level that was more than 10-fold larger than the previously reported common variant. 4 The study by Sabater-Lleal et al 5 identified an additional 11 gene associations with the VWF level, all in common variants and with modest effect sizes. Overall, the top variants for all loci independently associated with the level of VWF explained 21% of the variance in the VWF level.

Additional contributions to VWF level variation may come from rare variants that are not captured by the previously used marker arrays. To search for such variants, a population of VWD patients showing decreased levels of VWF was sequenced to screen comprehensively for genetic variation in the coding regions of the eight genes previously associated with VWF level variation. The underlying hypothesis is that these genes may harbor rare variants in addition to the common variants in some of these individuals with low VWF levels.

## Materials and Methods

### Study Population and von Willebrand Disease Phenotyping

The VWD study population was recruited from the Department for Coagulation Disorders, Malmö University Hospital (Malmö, Sweden). The population consists of consecutive patients and their relatives who have attended the clinic between the years 1988 and 2005, corresponding to approximately 1,000 individuals belonging to 127 families. This population represents the majority of all the families diagnosed with VWD in Sweden during this time period. Clinical and laboratory data were recorded for each patient, and their bleeding phenotypes were classified. 7 We used archival VWF levels usually determined at the time of the original diagnostic work-up. There were no further analyses of VWF levels in this study. Therefore, different phenotypical methods have been used. VWF activity was measured with the traditional VWF:RCo (ristocetin cofactor) method based on aggregation of platelets or an automated VWF:RCo assay based on the BCS coagulation analyzer (Siemens Healthcare Diagnostics, Marburg, Germany) using the BC von Willebrand reagent. VWF antigen levels (VWF:Ag) were measured using electroimmunoassay (the Laurell method), and IRMA (immunoradiometric assay), ELISA (enzyme-linked immunosorbent assay), or LIA (line immunoassay). Previous DNA sequencing identified type 2 VWD mutations in 20 patients among the 127 patients initially diagnosed with VWD. 8 Of the remaining 107 VWD patients, 104 were analyzed in this study. Of the 104 patients, 72 had a family history of bleeding in addition to their bleeding phenotypes. The remaining 32 patients were individual patients with bleeding and low VWF levels. In a strict sense, not all index cases fulfilled the modern definition of type 1 VWD, but at the time of diagnosis, their bleeding symptoms in combination with lowered VWF levels were interpreted as reflecting type 1 VWD. Two Swedish control populations were also analyzed: control population 1 (C _1_ ) consisting of 192 individuals from the general population 9 and control population 2 (C _2_ ) consisting of 288 unrelated male individuals with no history of bleeding from the general population. 10 This study was approved by the Ethics Committee of the Medical Faculty, Lund University, and the Swedish Data Inspection Board. Written informed consent was obtained from all patients. DNA from human whole blood was isolated using a Qiagen Blood DNA kit (Qiagen, Hilden, Germany), and DNA concentrations were determined by fluorometry using PicoGreen (Molecular Probes, Eugene, Oregon, United States).

### Single-Nucleotide Variant Genotyping

Both the VWD and the C _1_ and C _2_ control populations were genotyped for rs8176719 ( *ABO* ), rs1063857 ( *VWF* ), rs9390459 ( *STXBP5* ), rs4981022 ( *STAB2* ), rs2726953 ( *SCARA5* ), rs7978987 ( *STX2* ), rs7150240 ( *TC2N* ), and rs868875 ( *CLEC4M* ) using TaqMan assays. The genotyping was performed according to the manufacturer's protocol on a Bio-Rad CFX 384, with genotypes determined using the CFX Manager Software (Bio-Rad Laboratories Inc., Hercules, California, United States).

### Ion Torrent Sequencing

The primer sets were designed using Ion AmpliSeq Designer to include all exonic, 5′UTR (5′ untranslated region), and flanking intronic sequences ( http://www.ampliseq.com , pipeline version 2.2.1). The multiplex primer pools were optimized such that the primers for systems with low read depths in a specific pool were added to the other pool, excluding overlapping systems. The library preparation was achieved using the Ion AmpliSeq Library Kit 2.0 (Thermo Fisher Scientific, Waltham, Massachusetts, United States) according to the manufacturer's protocol. The amplicons were barcoded using Ion Xpress Barcode Adapters (Thermo Fisher Scientific). Purification of the library was achieved using Agencourt AMPure XP reagent beads (Beckman Coulter Inc., Brea, California, United States). Library amplification was performed, and further purification steps were achieved using Agencourt AMPure XP reagent beads before elution of the final library. The library concentrations were determined by capillary electrophoresis using a Fragment Analyzer (Advanced Analytical Technologies, Ankeny, Iowa, United States) and a High Sensitivity NGS Fragment Analysis Kit (Advanced Analytical Technologies). Library normalization was performed to a concentration of approximately 50 pM before being pooled together. An amplification reaction was prepared according to the manufacturer's protocol and transferred to an Ion PGM Hi-Q View reaction filter (Thermo Fisher Scientific) before emulsion polymerase chain reaction was performed on an Ion OneTouch 2 instrument (Thermo Fisher Scientific). Enrichment of the ion sphere particles was performed on an Ion OneTouch ES instrument using Dynabeads MyOne Streptavidin C1 beads (Thermo Fisher Scientific). The sequencing process was carried out on an Ion PGM sequencer (Thermo Fisher Scientific) using Ion 316 chip V2 (Thermo Fisher Scientific). This allowed simultaneous analysis of eight samples at the coverage presented in this study. The loaded chip was sequenced using a 400-bp sequencing protocol with 850 flows of single nucleotides.

### Bioinformatic Analysis

The generated raw data was processed by the Ion Torrent Suite Software v5.0.5 (Thermo Fisher Scientific). The sequences were aligned to the *Homo sapiens* hg19 reference genome and stored as BAM files. The frequency parameter for variant calling was set to 0.25, generating VCF files for each library containing single-nucleotide variants (SNVs), small insertions and deletions (indels), and putative mutations. The generated VCF files were merged and multiallelic sites split to create a database containing all called variants. Annotation of the database VCF file was achieved through the Ensembl Variant Effect Predictor, and each variant was classified following the American College of Medical Genetics and Genomics guidelines using VarSome. Evaluation of detected variants of each library was performed in parallel using MuCor: Mutation Aggregation and Correlation. The non-Finish European population of gnomAD (Genome Aggregation Database) and the SweGen population were used to compare allele frequencies. The Genotype Tissue Expression (GTEx) database was analyzed for changes in expression relating to the common variants associated with VWF level variation.

## Results

### Frequencies of Common Variants Associated with von Willebrand Factor Level

Several GWASs have identified and replicated a set of common variants associated with the VWF level in eight genes: *ABO* , *VWF* , *STXBP5* , *STAB2* , *SCARA5* , *STX2* , *TC2N* , and *CLEC4M* . 3 5 11 The *ABO* blood group gene showed by far the strongest effect on the VWF level in all previous studies. Since VWF levels of individuals with blood group O are reduced by 25% in comparison to non-O individuals, blood group O is a very strong contributor to low VWF levels and is also more common in type 1 VWD populations in comparison to type 2 VWD and normal populations. 12 In our VWD population, the null allele of *ABO* shows the strongest deviation from the expected allele frequency: 84% in the VWD population compared to 62, 60, and 63%, respectively, in the three control populations ( Table 1 ; Supplementary Table S1 ). This difference is highly significant, and it means that the O blood group is present in 71% of individuals compared to the Swedish national average of approximately 40% (refer to Supplementary Table S1 for details on all *ABO* alleles and genotypes). The VWF level associated alleles of the remaining seven genes were enriched by 2 to 7% in the VWD population compared to two local control populations of 192 and 288 individuals (C _1_ and C _2_ populations, respectively). They were also enriched compared to SweGen and gnomAD populations for six out of seven genes ( Table 1 ). However, the observed differences were not particularly large with enrichments between 1% and 7% for the different combinations of populations nor were they significant. Many of these variants are reported in the GTEx as being associated with changes in the expression level of the respective genes ( Table 1 and Supplementary Table S2 ). The genotyping produced data for all variants in >98% of individuals.

**Table 1:** Comparison of allele frequencies between VWD and local control (C _1_ and C _2_ ): SweGen and the non-Finnish European gnomAD populations

Gene	Variant	Risk allele frequency (%)									
		VWD	C _1_	C _2_	C _1 _ + C _2_	VWD-(C _1_ + C _2_ )	SweGen	VWD-SweGen	gnomAD	VWD-gnomAD	*P* -value a
*ABO* b	rs8176719 c	84	64	61	62	22	60	24	63	21	1.3 × 10 ^−9^
*VWF*	rs1063857	69	65	65	65	4	64	5	64	5	0.27
*STXBP5* b	rs9390459	51	45	47	46	5	45	6	44	7	0.19
*STAB2*	rs4981022	32	28	26	27	5	31	1	33	-1	0.14
*SCARA5* b	rs2726953	75	73	72	72	3	77	-2	72	3	0.38
*STX2* b	rs7978987	70	63	63	63	7	67	3	66	4	0.06
*TC2N* b	rs7150240 c	58	54	58	56	2	55	3	55	3	0.60
*CLEC4M* b	rs868875	37	31	36	34	3	30	7	32	5	0.40

Abbreviations: gnomAD, Genome Aggregation Database; VWD, von Willebrand disease; VWF, von Willebrand factor.

Note: common VWF level associated variants defined by Smith et al. 3

a Pearson's *χ* ^2^ test for independence comparing the VWD and C _1_ + C _2_ populations.

b Genes showing varying expression due to their respective variant as specified in Supplementary Table S2 .

c Variant used as proxy for the variant reported in Smith et al 3 ; rs8176719 has *R* ^2^ = 0.98 compared to rs687621, and rs7150240 has *R* ^2^ = 1 compared to rs10133762.

### Rare Variants in von Willebrand Factor Level Associated Genes

The coding sequences of *ABO* , *VWF* , *STXBP5* , *STAB2* , *SCARA5* , *STX2* , *TC2N* , and *CLEC4M* were screened for common and rare genetic variants by Ion Torrent sequencing of 104 patients from the historic VWD population. The patients in this population had bleeding symptoms in combination with lowered VWF levels, and we hypothesized that eventual rare variants with larger effects on the VWF level could be enriched in this population. The sequencing detected a total of 146 variants in the coding sequences of the eight genes. Excluding 70 variants in *VWF* (described in detail in the study by Manderstedt et al), 8 76 variants remained. There were a total of 19 variants in *ABO* : eight missense, two frameshift, and nine synonymous. Of the 19 variants, 15 had a minor allele frequency (MAF) of >5% in gnomAD, and the majority of these showed large allele frequency differences when comparing VWD and gnomAD populations, reflecting the large haplotype frequency differences present for *ABO* . Three of the four variants with MAFs < 5% in gnomAD were synonymous, and the fourth was a likely benign missense variant. Using PolyPhen2, three out of eight missense variants were predicted to be possibly or probably damaging ( Tables 2 and 3 ). They were all common variants with MAFs at ∼20%.

**Table 2:** Number of common and rare variants detected by targeted resequencing of 104 VWD patients

Gene	MAFs in gnomAD			
	<0.05%	0.05–0.5%	0.5–5%	>5%
*ABO*	2	0	2	15
*STXBP5*	0	0	4	2
*STAB2*	7	5	8	9
*SCARA5*	2	1	2	5
*STX2*	0	1	1	1
*TC2N*	1	1	2	0
*CLEC4M*	1	1	1	2

Abbreviations: gnomAD, Genome Aggregation Database; MAF, minor allele frequency.

Note: MAFs as reported for the non-Finnish European gnomAD population.

**Table 3:** Characteristics of variants detected in *ABO* , *STXBP5* , *STAB2* , *SCARA5* , *STX 2* , *TC2N* , and *CLEC4M* by targeted resequencing of 104 VWD patients and frequency comparisons with the non-Finnish European gnomAD population

Gene	SNP ID	Major/minor allele	MAF		LD to CV ( *R* ^2^ )	LD to CV ( *D* ')	Protein effect	VarSome (ACMG)	PolyPhen2	MutationTaster
			VWD	gnomAD						
*ABO*	rs139670895	C/T	0.48	0.00	Monoallelic, CEU	Monoallelic, CEU	Synonymous	VUS		
*ABO*	rs8176751	C/T	0.48	9.50	0.13	1.00	Synonymous	Benign		
*ABO*	rs56392308	–/GG	6.25	6.92	0.17	1.00	Frameshift	Benign		
*ABO*	rs8176749	C/T	0.48	7.78	0.13	1.00	Synonymous	Benign		
*ABO*	rs1474135537	G/A	0.48	0.00	–	–	Synonymous	VUS		
*ABO*	rs8176748	C/T	30.29	23.74	0.24	1.00	Missense	Benign	Prob. damaging	
*ABO*	rs8176745	G/A	30.77	23.91	0.24	1.00	Synonymous	Benign		
*ABO*	rs8176744	G/T	13.94	4.03	0.01	1.00	Synonymous	Benign		
*ABO*	rs8176743	C/T	0.96	7.65	0.13	1.00	Missense	Benign	Benign	
*ABO*	rs8176742	C/T	31.25	21.79	0.24	1.00	Synonymous	Benign		
*ABO*	rs8176741	G/A	0.96	7.75	0.13	1.00	Synonymous	Benign		
*ABO*	rs8176740	A/T	5.29	22.08	0.24	1.00	Missense	Benign	Prob. damaging	
*ABO*	rs7853989	G/C	0.96	9.87	0.13	1.00	Missense	Benign		
*ABO*	rs1053878	G/A	0.96	7.14	0.16	0.92	Missense	Benign		
*ABO*	rs8176720	T/C	32.21	33.71	0.06	0.41	Synonymous	Benign		
*ABO*	rs8176719	–/C	15.87	37.26	1.00	1.00	Frameshift	Benign		
*ABO*	rs512770	G/A	21.63	20.27	0.10	0.92	Missense	Benign	Poss. damaging	
*ABO*	rs688976	C/A	31.25	24.11	0.23	1.00	Missense	Benign	Benign	
*ABO*	rs8176696	C/T	1.44	1.84	0.00	1.00	Missense	Likely benign	Benign	
*STXBP5*	rs144099092	C/G	0.48	0.51	Monoallelic, CEU	Monoallelic, CEU	Missense	VUS	Benign	D
*STXBP5*	rs148830578	A/G	0.96	0.51	0.008	1	Missense	VUS	Benign	D
*STXBP5*	rs34324348	C/T	0.48	2.01	0.042	1	Synonymous	Likely benign		
*STXBP5*	rs34677388	A/G	2.4	2.41	0.027	1	Synonymous	Likely benign		
*STXBP5*	rs9390459	G/A	48.96	43.9	1	1	Synonymous	Benign		
*STXBP5*	rs1039084	A/G	47.6	45.88	0.92	0.979	Missense	Benign	Benign	P
*STAB2*	rs760013563	C/T	0.48	0	–	–	Synonymous	VUS		
*STAB2*	rs377067548	C/G	0.48	0	–	–	Synonymous	VUS		
*STAB2*	rs377011398	G/A	0.48	0	–	–	Missense	VUS	Benign	D
*STAB2*	12:104067708	T/C	0.48	0	–	–	Missense	VUS	Prob. damaging	D
*STAB2*	rs766516074	G/C	0.48	0.01	–	–	Missense	Likely benign	Benign	N
*STAB2*	rs189453513	C/G	0.48	0.03	Monoallelic, CEU	Monoallelic, CEU	Synonymous	Likely benign		
*STAB2*	rs142351376	C/T	0.96	0.03	Monoallelic, CEU	Monoallelic, CEU	Missense	Likely Benign	Benign	D
*STAB2*	rs151219602	C/T	0.48	0.05	Monoallelic, CEU	Monoallelic, CEU	Synonymous	Likely benign		
*STAB2*	rs149382223	T/C	0.48	0.18	0	0.008	Missense	Likely benign	Prob. damaging	D
*STAB2*	rs2271636	G/T	0.48	0.19	0.008	0.504	Synonymous	Benign		
*STAB2*	rs2271638	C/T	0.48	0.21	0.021	1	Synonymous	Benign		
*STAB2*	rs34699746	C/T	0.48	0.43	0.001	0.256	Synonymous	Likely benign		
*STAB2*	rs147053330	G/A	0.48	0.93	0.002	1	Synonymous	Likely benign		
*STAB2*	rs149524008	G/T	0.48	1.1	0.008	0.504	Missense	Likely benign	Benign	N
*STAB2*	rs116894406	G/A	0.48	1.25	0.004	0.492	Missense	Likely benign	Poss. damaging	D
*STAB2*	rs148397037	G/A	1.44	1.56	0.001	0.256	Synonymous	Likely benign		
*STAB2*	rs17034336	G/A	0.96	1.9	0.001	0.107	Missense	Benign	Benign	N
*STAB2*	rs7973658	G/A	0.96	2.08	0	0.008	Missense	Benign	Benign	N
*STAB2*	rs17034433	A/C	0.96	3.33	0.02	0.766	Missense	Benign	Benign	P
*STAB2*	rs35102665	G/A	3.85	3.86	0.015	1	Synonymous	Likely benign		
*STAB2*	rs7306642	C/A	5.77	6.41	0.006	0.391	Missense	Benign	Benign	P
*STAB2*	rs1609860	C/A	8.17	8.54	Triallelic	Triallelic	Missense	Benign	Prob. damaging	P
*STAB2*	rs2292688	C/T	12.02	10.44	0.009	0.365	Synonymous	Benign		P
*STAB2*	rs10778281	C/T	10.58	13.69	0	0.023	Synonymous	Benign		
*STAB2*	rs2271637	C/G	25.96	25.91	0.154	0.941	Missense	Benign	Poss. damaging	P
*STAB2*	rs697210	T/C	31.25	28.58	0	0.019	Synonymous	Benign		
*STAB2*	rs11614418	C/T	38.46	37.25	0.016	0.249	Synonymous	Benign		P
*STAB2*	rs703651	C/T	40.87	44.07	0.02	0.222	Synonymous	Benign		
*STAB2*	rs697212	T/C	48.08	48.8	0.002	0.076	Synonymous	Benign		
*SCARA5*	rs17058374	C/T	0.48	0	Monoallelic, CEU	Monoallelic, CEU	Missense	Likely benign	Benign	N
*SCARA5*	rs61737292	G/A	0.48	0.02	Monoallelic, CEU	Monoallelic, CEU	Missense	Benign	Benign	D
*SCARA5*	rs148420420	C/T	0.48	0.06	–	–	Synonymous	VUS		
*SCARA5*	rs61737296	G/A	0.48	1.21	0.007	1	Synonymous	Likely benign		
*SCARA5*	rs118119884	G/A	2.88	3.53	0.009	1	Missense	Benign	Benign	N
*SCARA5*	rs6983356	G/A	9.13	9.23	0	0.021	Synonymous	Benign		
*SCARA5*	rs17058207	C/G	10.58	9.67	0.029	0.718	Missense	Benign	Benign	P
*SCARA5*	rs61737291	G/A	12.98	12.04	0.015	0.619	Synonymous	Benign		
*SCARA5*	rs4732610	G/A	13.94	13.77	0	0.017	Synonymous	Benign		
*SCARA5*	rs10103504	T/C	39.71	38.35	0.549	0.969	Synonymous	Benign		
*STX2*	rs145834567	T/C	0.48	0.08	Monoallelic, CEU	Monoallelic, CEU	Missense	VUS	Poss. damaging	D
*STX2*	rs137928907	A/C	2.88	2.57	0.016	1	Missense	VUS	Poss. damaging	D
*STX2*	rs17564	G/C	30.29	34.87	0.978	1	Missense	Benign	Benign	P
*TC2N*	rs756737332	C/T	0.48	0.01	–	Missense	Missense	VUS	Benign	N
*TC2N*	rs144110131	T/G	0.48	0.39	Monoallelic, CEU	Monoallelic, CEU	Missense	VUS	Benign	D
*TC2N*	rs2402073	G/T	2.88	2.46	Triallelic	Triallelic	Missense	Benign	Benign	N
*TC2N*	rs2402073	G/A	2.88	2.46	Triallelic	Triallelic	Missense	Likely benign	Benign	N
*CLEC4M*	rs753084254	C/T	0.48	0	–	–	Synonymous	VUS		
*CLEC4M*	rs113080783	A/G	3.85	0.08	–	–	Missense	VUS	Benign	N
*CLEC4M*	rs62623420	A/G	0.48	0.51	0.004	1	Splice acceptor	VUS		
*CLEC4M*	rs868878	G/A	14.42	13.16	0.057	1	Synonymous	Benign		
*CLEC4M*	rs2277998	G/A	36.42	31.61	1	1	Missense	Benign	Benign	P

Abbreviations: ACMG, American College of Medical Genetics and Genomics; CV, common variant; D, deleterious; gnomAD, Genome Aggregation Database; LD, linkage disequilibrium; MAF, minor allele frequency; N, neutral; P, polymorphism; poss., possibly; prob., probably; SNP, single-nucleotide polymorphism; VUS, variant with uncertain significance.

The remaining six genes contained a total of 57 variants. The numbers of common and rare variants detected for the individual genes are shown in Table 2 . Twenty-nine variants were detected in *STAB2* , a clearly higher number of variants compared to the other five genes, which had between 3 and 10 variants. Of the 57 variants, 19 had an MAF of >5% and 20 variants had an MAF of <0.5% in gnomAD. There were almost equal numbers of synonymous and missense variants (27 and 29, respectively). Using VarSome to predict the function of the 57 variants, an absolute majority (43) were found to be benign or likely benign, whereas only 14 were predicted to be of uncertain significance, and none was predicted as pathogenic ( Table 3 ). When using PolyPhen2 instead, 7 out of 29 missense variants were predicted to be possibly or probably damaging. Of these, five were in *STAB2* and two in *STX2* . No variants had significantly different MAFs when compared between VWD and gnomAD populations. All tests for accumulation of low frequency (0.05%-0.5%) or rare variants (<0.05%) in the VWD population compared to the gnomAD population were insignificant ( Table 4 ). To investigate for Swedish-specific allele frequencies, SweGen and gnomAD populations were compared for their numbers of low frequency or rare variants with an insignificant result ( Supplementary Table S3 ).

**Table 4:** Fisher's exact test for accumulation of low frequency and rare variant alleles by comparison between VWD and the non-Finnish European gnomAD populations

Gene	MAF < 0.05%				*p* -Value	MAF 0.05-0.5%				*p* -Value
	VWD		gnomAD			VWD		gnomAD		
	Alleles	Chr	Alleles	Chr		Alleles	Chr	Alleles	Chr	
*ABO*	2	208	416	64417	0.40	0	208	1132	64542	1.0
*STXBP5*	0	208	928	105841	1.0	0	208	985	118481	1.0
*STAB2*	7	208	4470	110794	0.74	5	208	5015	117518	0.94
*SCARA5*	2	208	650	95809	0.41	1	208	468	112869	0.58
*STX2*	0	208	308	105084	1.00	1	208	371	120076	0.48
*TC2N*	1	208	511	125998	0.57	1	208	494	125998	0.56
*CLEC4M*	1	208	544	110222	0.64	1	208	873	124390	0.77

Abbreviations: gnomAD, Genome Aggregation Database; MAF, minor allele frequency; VWD, von Willebrand disease.

### Read Depth and Coverage

The eight genes were analyzed using an AmpliSeq panel targeting all exons and flanking intronic regions. The coverage was close to 100% for all genes except for *ABO* and *STX2* , where single exons were missing due to low-yielding primer systems, and *TC2N* , where a single exon was missing due to a failed primer design ( Supplementary Fig. S1 ). All variants with MAFs > 5% in gnomAD were detected in this study, except for single variants in each of *STX2* and *TC2N* that were in the missing exons of those genes. The average read depth varied between 176 and 570, with a read depth of >300 for seven of the eight genes ( Supplementary Table S4 ; Supplementary Fig. S1 ). The average strand bias was <10% for all genes. All reported low frequency and rare variants were supported by >100 reads. The coding sequences of exons 1 to 3 and exons 5 to 7 of *CLEC4M* had been sequenced previously using Sanger sequencing, 13 as had all exons of *STXBP5* . 14 There was complete concordance between the sets of variants detected by Sanger and Ion Torrent sequencing for all individuals.

## Discussion

This study investigated 104 patients from a historic VWD population with lowered VWF levels for common and rare variants in *ABO* , *VWF* , *STXBP5* , *STAB2* , *SCARA5* , *STX2* , *TC2N* , and *CLEC4M* genes. Common variants in these genes had initially been reported by Smith et al 3 to be associated with the VWF level in a large GWAS and we hypothesized that the VWD patients in this study would show the same associations. This was indeed the case, as all eight VWF level associated alleles were enriched in the VWD population compared to two local control populations and for seven out of eight genes compared to SweGen and gnomAD populations. The observed allele frequency enrichments for the three combinations of populations were largest for the *ABO* variant, with an enrichment of approximately 20%. The enrichments detected for the remaining seven genes were considerably smaller, varying between 2 and 7% for the local control populations. The allele frequency difference detected for the *ABO* variant was significant, whereas differences detected for the other variants were not. These results are comparable with the ones reported by Sanders et al, 15 who found a similar pattern of enrichment in their VWD population. This indicates that there is a general enrichment of these GWAS-defined VWF level associated alleles among type 1 VWD patients or low VWF individuals and that our VWD population also shows this pattern.

The Ion Torrent sequencing of *ABO* , *VWF* , *STXBP5* , *STAB2* , *SCARA5* , *STX2* , *TC2N* , and *CLEC4M* in 104 patients from our historic VWD population detected a total of 146 variants in the coding sequences of the eight genes. *VWF* harbored 70 of these, of which many were mutations and have been described in detail previously. 8 Of the remaining 76 variants, 54 had allele frequencies > 0.5% and a majority of these have therefore likely already been investigated for their association with the VWF level in later GWAS also investigating low-frequency variants in these and other genes. 4 6 The rs141041254 *STAB2* variant associated with VWF level variation and identified by Huffman et al 4 was not found in this study, but it has been further investigated along with common variants in *STAB2* . 16 The 22 variants with frequencies < 0.5% are less likely to have been previously evaluated in the present context, even though all but one is present in gnomAD. According to VarSome, 12 of these had an uncertain significance and 10 were benign or likely benign. PolyPhen2 classified 3 out of the 22 variants as probably or possibly damaging (two in *STAB2* and one in *STX2* ); the others were either synonymous or denoted benign. In comparison, MutationTaster classified 7 out of the 22 variants as deleterious. In addition, the testing for accumulation of low frequency or rare variants in the VWD population compared to gnomAD was not significant for any of the seven genes. We have therefore rejected our hypothesis that rare variants with larger effects on the VWF level are accumulated in these genes, and even if a few of the rare variants detected in this study really have an effect on the VWF level, their overall contribution to disease in the VWD population must be very limited indeed.

Our study should be interpreted within the context of its limitations. First, our study population is rather small, thereby limiting the absolute number of chromosomes interrogated for rare variants and consequently also limiting our ability to detect rare variants affecting the VWF level. Our population consists of a mixture of type 1 VWD patients and patients with a low VWF phenotype who do not fulfill modern criteria for type 1 VWD. However, the selection of individuals without causal *VWF* mutations, but with low VWF levels, may rather increase the likelihood of finding rare variants affecting VWF level in those individuals. Second, the absence of detected rare variants in this study may be caused by an inability of our sequencing methodology to detect variants that are present in the population. We would like to argue against this since the average read depths were >300 for seven of the eight genes, the coverage was high with only very few regions without coverage, and there was a complete concordance between the sets of variants detected by Sanger and Ion Torrent sequencing in *CLEC4M* and *STXBP5* for all individuals. 13 14 Thus, we believe that the lack of accumulation of rare variants and the almost total absence of rare variants predicted to affect protein function in our VWD population is true. None of the genes, except the *VWF* gene itself, harbor large numbers of rare variants affecting the amount of VWF, ultimately giving rise to VWD. Obviously, we cannot completely exclude the fact that there might exist single rare variants in any of these genes that can have a major effect on the VWF level, but we think it is reasonable to argue that a large number of such variants do not exist.

This leaves us with the following explanatory model for the VWF level variation achieved by genetic variants in the eight genes *.* The *VWF* itself harbors both a large number of *bona fide* mutations and a common haplotype containing SNVs c.2365A > G and c.2385T > C affecting VWF biosynthesis and clearance. 2 17 The rs1063857 (c.2385T > C) variant analyzed in this study and the rs1063856 (c.2365A > G) are reported to be in strong linkage disequilibrium and influence VWF levels independently. 17 The low VWF allele of rs1063857 was enriched in our VWD population by approximately 5% compared to all three control populations, which is in agreement with previous reports. Individuals with different ABO blood groups have different VWF levels. Individuals with AB blood group have a longer VWF half-life compared to individuals with O blood group, who have on average of approximately 25% lower VWF level compared to non-O blood group individuals. More complex antigens cause VWF to be less susceptible to clearance from the circulation. 18 In this study, the null allele of *ABO* showed the strongest deviation from the expected allele frequency: 84% in the VWD population compared to 62, 60, and 63%, respectively, in the three control populations. No contribution from rare variants could be detected for *ABO* in our VWD population since only two synonymous variants were detected with MAFs < 0.5%.

*STXBP5, STX2* , and *TC2N* are most likely involved in the synthesis and exocytosis of VWF from endothelial cells. Thus, it is likely that common variants associated with a decreased VWF level are either associated with lower expression levels giving rise to fewer of these protein molecules or else they hamper the function of the proteins. In addition, the GTEx database reports that the VWF-associated common alleles in these genes are indeed associated with decreased expression levels of all three of the investigated genes, which is compatible with our observations in the VWD population. It is likely that common and rare variants would work in the same direction in these genes. Rare loss-of-function alleles would therefore be accumulated among patients with a bleeding phenotype. Previous analysis of *STXBP5* revealed two benign rare variants and a common missense variant (rs1039084) with a slightly increased allele frequency compared to controls. 14 *STX2* had two rare missense variants, which were possibly damaging, and one common missense variant, which was benign. *TC2N* had four benign missense variants with the same allele frequencies as the control populations. Thus, none of these genes show accumulation of rare variants with damaging effects.

*STAB2, SCARA5* , and *CLEC4M* are receptors that operate through binding and eliminating VWF in the bloodstream. It is likely that the more of these molecules and the more effectively they bind VWF, the lower the VWF levels will be. This means that common variants associated with the VWF level likely function through more effective binding or higher expression levels giving rise to more of these proteins. Rare mutations within these genes would have to be of a gain-of-function type to produce the phenotype typical of VWD. Since this type of mutation appears much less frequently than loss-of-function type mutations, it is highly unlikely that there would be an accumulation of such mutations. The VWF level association observed for *CLEC4M* was speculated to depend either on a missense mutation (rs2277998) or on the existence of heterozygosity for the tandemly repeated sequence present in exon 4. 13 Two rare variants were observed in the VWD population: one synonymous and one benign missense variant. Neither is a likely contributor to a low VWF phenotype. The common variant of *SCARA5* reported by Smith et al 3 was only slightly enriched in our VWD population compared to two of the control populations. This gene had two benign missense variants and one synonymous rare variant and showed no allele frequency differences for the remaining seven variants. Excluding *VWF* , *STAB2* was the most variable of the genes investigated in this study. It had five probably or possibly damaging rare missense variants but did not show any accumulation of rare variants compared to gnomAD. Since this gene shows a lot of variation and seems to be tolerant to changes in its protein sequence, these missense variants are also less likely to contribute to changes in VWF level and ultimately to VWD. In conclusion, we do not find any support for our hypothesis that rare variants are accumulated in the VWD population and contribute to the disease phenotype. In addition, the few rare variants detected in these genes are not likely to affect protein function. The common variants previously associated with the VWF level all show the expected pattern in our VWD population, indicating that the population as such is valid to use for the purpose of rare variant analysis, as described previously.
