# Supplementary material for: Common and Rare Variants in Genes Associated with von Willebrand Factor Level Variation: No Accumulation of Rare Variants in Swedish von Willebrand Disease Patients
Source: TH Open. 2020 Oct 31;4(4):e322–31. doi: 10.1055/s-0040-1718885 (PMC7603419; doi:10.1055/s-0040-1718885)
Supplement: Supplementary file 1 — Supplementary Material [file 10-1055-s-0040-1718885-s200047.pdf]

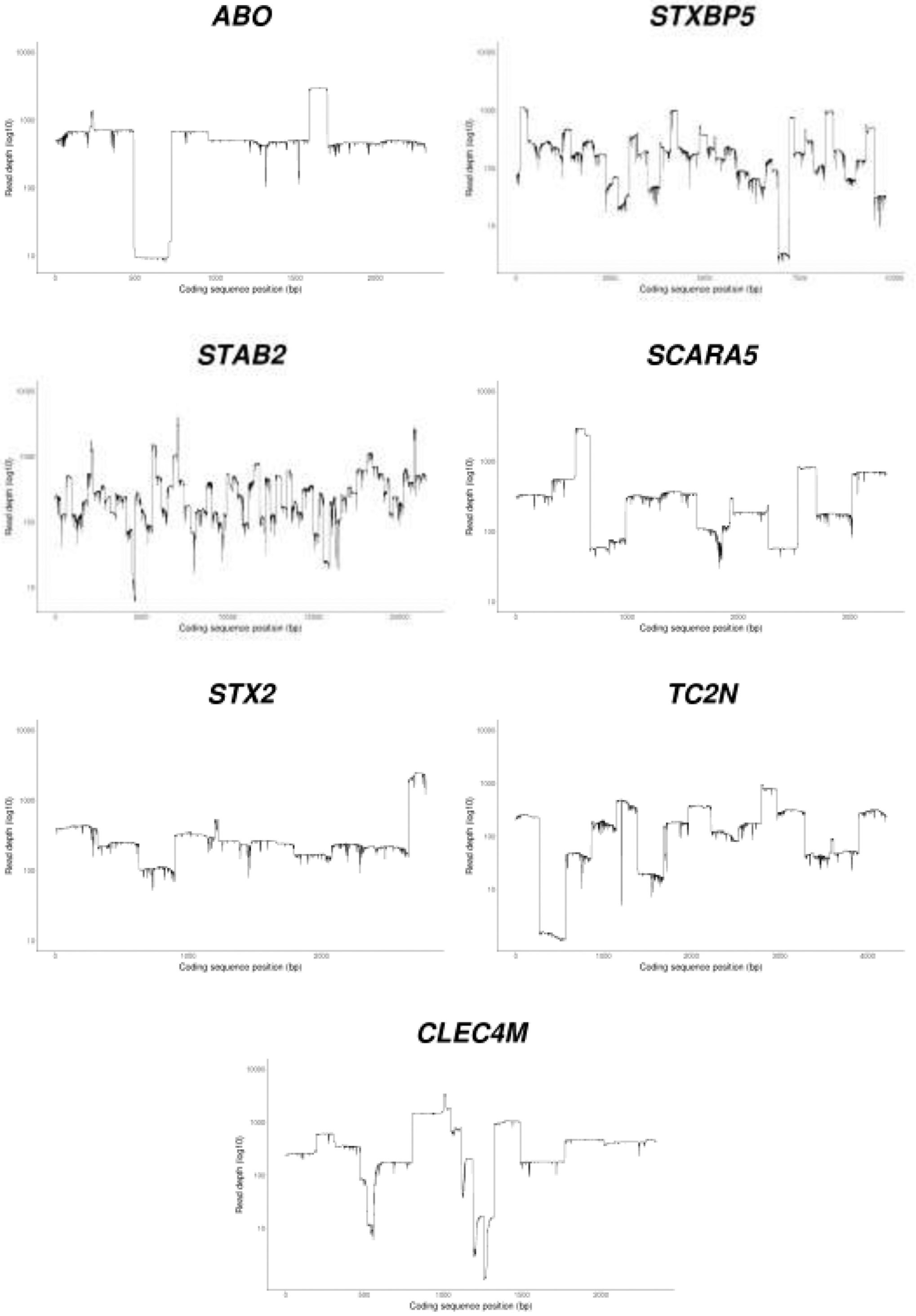

Supplementary Fig. S1 Mean read depth for *ABO*, *STXBP5*, *STAB2*, *SCARA5*, *STX2*, *TC2N*, and *CLEC4M*.

Supplementary Table S1 Blood group alleles and genotypes in the VWD population

ABO allele	No. of alleles	%
O	174	84
A	20	10
A2	13	6
B	1	0
ABO genotype	No. of Individuals	%
O/O	74	71
O/A	14	13
O/A2	11	11
O/B	1	1
A/A	3	3
A2/A2	1	1

Abbreviation: VWD, von Willebrand disease.

Supplementary Table S2 Expression variation as detailed in GTEx database for genes associated with VWF level variation

Gene Symbol	SNP ID	REF allele	ALT allele	*p*-Value	NES[a]	Tissue
ABO	rs8176719	T	TC	1.70E-86	0.8	Muscle: skeletal
ABO	rs8176719	T	TC	9.00E-73	0.75	Adipose: visceral (omentum)
ABO	rs8176719	T	TC	9.50E-64	0.67	Esophagus: mucosa
ABO	rs8176719	T	TC	3.10E-48	0.55	Heart: left ventricle
ABO	rs8176719	T	TC	1.80E-40	0.43	Lung
STXBP5	rs9390459	A	G	7.30E-08	0.18	Brain: cortex
STXBP5	rs9390459	A	G	0.0000052	0.094	Esophagus: mucosa
STXBP5	rs9390459	A	G	0.000014	0.14	Brain: frontal cortex (BA9)
STXBP5	rs9390459	A	G	0.0001	0.048	Whole Blood
SCARA5	rs2726953	G	A	6.30E-12	−0.65	Spleen
SCARA5	rs2726953	G	A	1.10E-11	−0.18	Adipose: subcutaneous
SCARA5	rs2726953	G	A	7.80E-07	−0.38	Ovary
SCARA5	rs2726953	G	A	0.0000045	−0.13	Nerve: tibial
SCARA5	rs2726953	G	A	0.000011	−0.14	Adipose: visceral (omentum)
STX2	rs7978987	G	A	6.10E-63	−0.56	Whole blood
STX2	rs7978987	G	A	8.30E-48	−0.62	Lung
STX2	rs7978987	G	A	5.10E-42	−0.45	Skin: not sun exposed (suprapubic)
STX2	rs7978987	G	A	9.30E-39	−0.5	Adipose: visceral (omentum)
STX2	rs7978987	G	A	7.60E-38	−0.48	Adipose: subcutaneous
TC2N	rs7150240	T	C	1.50E-55	−0.62	Nerve: tibial
TC2N	rs7150240	T	C	1.90E-48	−0.54	Adipose: subcutaneous
TC2N	rs7150240	T	C	7.80E-38	−0.46	Muscle: skeletal
TC2N	rs7150240	T	C	3.00E-37	−0.32	Esophagus: mucosa
TC2N	rs7150240	T	C	9.40E-36	−0.53	Esophagus: muscularis
CLEC4M	rs868875	A	G	2.60E-34	−0.79	Testis
CLEC4M	rs868875	A	G	3.30E-13	−0.63	Brain: cortex
CLEC4M	rs868875	A	G	1.90E-12	−0.64	Brain: frontal cortex (BA9)
CLEC4M	rs868875	A	G	3.80E-12	−0.58	Brain: cerebellum

Abbreviations: ALT, alternative allele; GTEx, Genotype Tissue Expression; SNP, single-nucleotide polymorphism; REF< reference allele; VWF, von Willebrand factor.
Note: the table shows up to the five most significant types of tissues for the common variants previously associated with VWF level variation.[3]
[a]NES, the slope of the linear regression, is computed as the effect of the ALT relative to the REF in the human genome reference (i.e., the eQTL effect allele is the ALT allele). NES are computed in a normalized space where magnitude has no direct biological interpretation.

Supplementary Table S3 Fisher's exact test for accumulation of low frequency and rare variant alleles: comparison of SweGen and the non-Finnish European gnomAD populations

Gene	MAF $<$ 0.05%				*p*-Value	MAF 0.05-0.5%				*p*-Value
	SweGen		gnomAD			SweGen		gnomAD		
	Alleles	Chr	Alleles	Chr		Alleles	Chr	Alleles	Chr	
ABO	8	2000	416	64417	0.94	21	2000	1132	64542	0.99
STXBP5	14	2000	928	105841	0.83	14	2000	985	118481	0.77
STAB2	38	2000	4470	110794	1.00	75	2000	5015	117518	0.9
SCARA5	8	2000	650	95809	0.96	10	2000	468	112869	0.32
STX2	3	2000	308	105084	0.93	5	2000	371	120076	0.74
TC2N	10	2000	511	125998	0.30	4	2000	494	125998	0.95
CLEC4M	5	2000	544	110222	0.97	9	2000	873	124390	0.94

Abbreviations: gnomAD, Genome Aggregation Database; MAF, minor allele frequency; VWD, von Willebrand disease.

Supplementary Table S4 Mean read depth

Gene	Read depth	Strand bias	Error frequency
ABO	570	0.48	0.0005
STXBP5	302	0.51	0.0005
STAB2	366	0.49	0.0005
SCARA5	438	0.52	0.0006
STX2	348	0.48	0.0005
TC2N	176	0.47	0.0005
CLEC4M	487	0.47	0.0006
